# Cell-Based Therapy and Genome Editing as Emerging Therapeutic Approaches to Treat Rheumatoid Arthritis

**DOI:** 10.3390/cells13151282

**Published:** 2024-07-30

**Authors:** Vitaly Chasov, Irina Ganeeva, Ekaterina Zmievskaya, Damir Davletshin, Elvina Gilyazova, Aygul Valiullina, Emil Bulatov

**Affiliations:** 1 Laboratory of Biomedical Technologies, Institute of Fundamental Medicine and Biology, Kazan Federal University, 18 Kremlyovskaya Street, Kazan 420008, Russia; 2 Shemyakin-Ovchinnikov Institute of Bioorganic Chemistry, Russian Academy of Sciences, Moscow 117997, Russia; 3 Federal State Autonomous Educational Institution of Higher Education I.M. Sechenov First Moscow State Medical University of the Ministry of Health of the Russian Federation (Sechenov University), Moscow 119048, Russia

**Keywords:** rheumatoid arthritis, microRNA, CRISPR-Cas9, mesenchymal stem cell, CAR-T cells

## Abstract

Rheumatoid arthritis (RA) is an autoimmune disease characterized by chronic inflammation of the joints. Although much remains unknown about the pathogenesis of RA, there is evidence that impaired immune tolerance and the development of RA are related. And it is precisely the restoration of immune tolerance at the site of the inflammation that is the ultimate goal of the treatment of RA. Over the past few decades, significant progress has been made in the treatment of RA, with higher rates of disease remission and improved long-term outcomes. Unfortunately, despite these successes, the proportion of patients with persistent, difficult-to-treat disease remains high, and the task of improving our understanding of the basic mechanisms of disease development and developing new ways to treat RA remains relevant. This review focuses on describing new treatments for RA, including cell therapies and gene editing technologies that have shown potential in preclinical and early clinical trials. In addition, we discuss the opportunities and limitations associated with the use of these new approaches in the treatment of RA.

## 1. Introduction

Rheumatoid arthritis (RA) is an autoimmune disease of complex etiology characterized by chronic inflammation of the synovial joints, primarily small peripheral joints, that can lead to heart, lung, or nervous system disorders, impairment of other vital body functions, and severe disability [1]. RA is more common in women than men and is estimated to affect 0.5–1% of the world’s population [2]. The prevalence of the disease and its importance to the health care system is illustrated by the fact that it affects approximately 1.3 million people in the United States [3]. The disease is associated with abnormalities in the function of immune cells such as lymphocytes and macrophages, along with abnormal activation of non-immune cells such as fibroblast-like synoviocytes and osteoclasts, which also contribute to synovial inflammation and joint degeneration [1]. Commonly accepted immunologic indicators for diagnosing RA in vivo are high levels of autoantibodies that react as an autoantigen with self-immunoglobulin G, called rheumatoid factor (RF), and autoantibodies to cyclic citrullinated peptide or type II collagen (CII) [4,5]. The planned treatment program includes monitoring progress after medication is prescribed through regular patient assessments. Achieving a state of sustained remission with a minimum of adverse side effects is the ideal goal of therapeutic intervention.

Traditional treatments include classical synthetic disease-modifying antirheumatic drugs (DMARDs) such as methotrexate, non-steroidal immunosuppressants, and glucocorticoids [6]. Therapy with these drugs requires special attention because they cause significant side effects, although under favorable circumstances they can reduce pain and inflammation, reduce joint damage, and slow the progression of the disease [2]. In addition, it has been found that a certain subgroup of patients have a partial or no response to conventional treatments. For example, 30% of patients have an inadequate response to methotrexate [7]. At the present time, there are also alternative medicines available, which are the result of many years of research. Biologic DMARDs and synthetic molecules such as Janus kinase (JAK) inhibitors are the next group of drugs, targeted therapies. These therapies have significantly changed the treatment of RA by specifically targeting the molecules involved in the inflammatory process and have shown promising results [8]. However, even targeted drugs are known to have certain drawbacks and limitations in use. A serious disadvantage of these drugs is their significantly higher price compared to traditional DMARDs, which is especially noticeable with long-term use [9]. JAK inhibitors have been reported to be most commonly associated with infectious adverse events, embolism and thrombosis, neoplasia and gastrointestinal perforation events, and hepatotoxicity and skin reactions [10]. The main side effect of biological agents, antibodies, is immunogenicity, i.e., the production of antidrug antibodies, especially against chimeric antibodies [11]. To reduce immunogenicity, combining antibodies with conventional DMARDs such as methotrexate is recommended [12]. Biologic DMARDs are also associated with an increased risk of serious infections due to their immunosuppressive effects compared to conventional DMARDs [13,14]. The efficacy of biologic DMARDs (mainly anti-tumor necrosis factor (TNF) therapy) has also been found to vary dramatically from patient to patient [15]. This variability has been suggested to be associated with a specific genetic background of RA onset, and the identification of genetic polymorphisms that have a significant impact on treatment response in RA patients would improve the management of this disease [16]. In addition, an analysis of the Surveillance, Epidemiology, and End Results (SEER)-Medicare data found that anti-TNF therapy increased the risk of certain cancers in older RA patients, particularly follicular lymphoma and the non-melanoma skin cancer (NMSC) and non-Hodgkin’s lymphoma (NHL) groups [17]. A meta-analysis of trial databases also showed an increased risk of cancer after anti-TNF treatment in RA patients [18].

Therefore, in addition to the above, there is a need to develop and use safer and more effective treatments for RA, ideally accessible to the general population at an affordable price. And with varying degrees of success, this search and development of alternative treatments for RA continues. In this review, we will take a look at some of the methods that have been shown to be promising, and discuss the need for, and possibility of, their use.

## 2. Cell-Based and Genome Editing Approaches in RA Therapy

**Figure 1:**
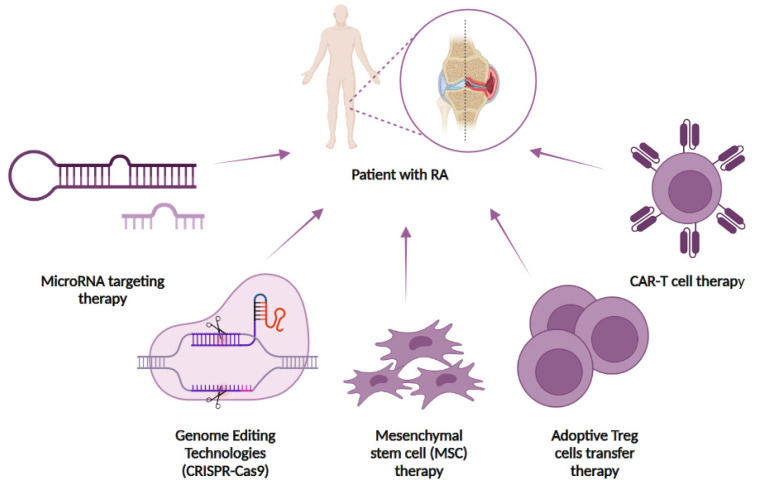
Potential novel strategies for targeting RA. Cell-based and genome-editing therapies for the treatment of RA include microRNA therapy, genome-editing using the CRISPR-Cas9 system, mesenchymal stem cell therapy, adoptive Treg cell transfer, and CAR T-cell therapy.

The scientific literature describes several approaches to the treatment of RA and other autoimmune diseases based on modern ideas about the nature, genetics, and pathophysiology of the disease. In the following, we will look at some of these new approaches, which seem to be the most attractive in terms of the idea and the first results obtained (Figure 1).

### 2.1. MicroRNAs Targeting Therapy

MicroRNAs (miRNAs) are small endogenous non-coding RNAs, averaging 22 nucleotides in length, that are involved in the post-transcriptional regulation of gene expression. MicroRNAs play an important role in the regulation of the immune response. They influence the proliferation, differentiation, maturation, and activation of immune cells, as well as the release of inflammatory mediators and the secretion of antibodies. When this regulation is disrupted, various pathological conditions of autoimmune inflammation can occur, including the development of RA [19]. Specific miRNA species may increase or decrease RA risk and disease activity (Table 1) [20,21,22,23]. In addition, microRNAs have been implicated not only in immune cell proliferation and differentiation, but also in synovial cell proliferation and apoptosis, synovial inflammation, and cartilage destruction in the pathogenesis of RA [24,25,26,27,28,29]. Experiments in rat models and in the mouse model of collagen-induced arthritis (CIA), the most convenient and widely used model of rheumatoid arthritis, showed that miRNAs can be therapeutic targets in RA fibroblast-like synoviocytes (RA-FLS) [29,30,31,32,33].

Serum levels of miR-181a, which regulate myotubularin-related protein 3 (MTMR3), an autophagy-related gene, were found to be positively associated with RA severity [34]. Levels of miR-25 and miR-378d were significantly down-regulated, whereas miR-371b, miR-483, and miR-642b were up-regulated in peripheral blood monocytes (PBMCs) from patients who had already developed RA [35]. These data correlated with elevated levels of IL-15, a monocyte-related factor, in the serum of patients [35]. These observations are important because high levels of IL-15 are associated with the progression of joint destruction in RA and are found even in preclinical patients [36,37]. At the same time, inhibiting IL-15 improves disease symptoms in animal models and patients [38]. The levels of miR-23 and miR-223 were found to be increased and at the same time TNFα, IL-6, and IL-17 levels were decreased after anti-TNFα/DMARDs therapy [39]. This observation suggests miR-23 and miR-223 as potential novel biomarkers for predicting and monitoring the outcome of RA therapy [39]. Another study showed significantly increased levels of miR-19b-3p in the sera of RA patients compared to controls, with levels of miR-19b-3p almost equal to healthy controls after treatment with baricitinib [40]. These data suggest that circulating miR-19b levels may be a promising novel biomarker for predicting joint inflammation and response to JAK inhibitors in RA patients [40].

The hsa-miR-21-5p has been implicated in the modulation of bone and joint homeostasis through the regulation of fibroblast-like synoviocytes, chondrocytes, and osteocytes, and is therefore of great importance in RA [41]. In addition, by reducing Stat3 activity, hsa-miR-21-5p inhibits the reprogramming of anti-inflammatory Tregs into pro-inflammatory cells secreting IL-17 [42]. Taking into account that the balance between Treg/Th17 turns out to be crucial during the course of RA, it was hypothesized that hsa-miR-21-5p could serve as a candidate for target therapy, since blocking hsa-miR-21-5p activity can restore the Treg/Th17 balance [43]. It has been suggested that miR-155 may also be a novel therapeutic target for the treatment of RA, as its deficiency prevented the formation of autoreactive T and B cells and led to impaired Th17 cell polarization in the CIA model [44]. Another candidate to be targeted in the treatment of RA is miR-340-5p, as its induction has been shown to prevent the proliferation of fibroblast-like synoviocytes (FLS) by targeting the transcription factor STAT3 [45]. The advantage of miRNA-targeting approaches is that miRNAs can simultaneously target multiple effectors of pathways involved in RA pathogenesis (Table 1). Several approaches have been proposed to block microRNA activity in clinical practice, the main ones being single-stranded antisense oligonucleotides, small interfering RNAs, locked nucleic-acid-based oligonucleotides, and small molecule inhibitors [46,47,48,49].

Thus, there is reason to hope that in the near future there will be a market for drugs targeting microRNAs that will occupy their niche as therapeutic agents for the treatment of RA and be used in clinical practice after appropriate preclinical and clinical trials.

### 2.2. Genome Editing Technologies

The Clustered Regularly Interspaced Short Palindromic Repeats (CRISPR)-Cas9 system is an RNA-guided bacterial adaptive immune system against bacteriophages and plasmid transfer [50]. CRISPR loci are short palindromic repeats (25–35 bp each) separated by small unique sequences called spacers (30–40 bp each). CRISPR loci are accompanied by CRISPR-associated (Cas) genes that encode Cas proteins [51,52]. When used for research purposes in the laboratory, this technology allows precise editing of various genes, opening up tremendous opportunities in clinical practice for the treatment of diseases associated with genomic changes, including RA. The possibility of using gene-editing technology to treat RA is based on extensive genetic studies that have attempted to identify regions of the genome associated with the onset of the disease [53,54,55,56,57]. In the early 2000s, the International HapMap Project presented the map of human genome sequence variations in multiple populations of Africa, Asia and Europe, suggesting the indirect association approach to any functional gene in the genome [55]. This approach later enabled genome-wide screening of genetic variants across the genome [mainly represented as single nucleotide polymorphisms (SNPs)] to associate them with disease risk factors [58,59]. Then, a new method, large-scale genome-wide association study (GWAS) using commercial microchips, was used for a wide range of complex human traits to determine the risk of predisposition to autoimmune diseases, including RA [54,56,57]. Then, meta-analyses of multiple GWASs identified a number of new RA risk genes [53,60,61]. There has also been collaboration and data sharing in the community of researchers of genetic characteristics of RA.

With the advent of such data, however, difficulties in its interpretation became apparent. One of the major challenges in current RA genetic research is the association of statistical data with specific cellular or molecular phenotypes [62]. In existing genetic studies, associated SNPs have been annotated by physical overlap with a specific gene or a neighboring gene [62]. However, increasing knowledge of the human genome architecture indicates that this type of signal annotation is often inaccurate and sometimes misleading [63,64]. Annotation requires a more comprehensive assessment of the function of genes that are sometimes located relatively far from the location indicated by the association analysis. One of the reasons for this is the structure of chromosomes, the so-called linked inheritance of genes. With the development of single-cell research platforms, much more specific and reliable annotation of such data is expected [62].

To date, GWASs and meta-analyses have identified common disease-associated variants in the global population that may cumulatively contribute to the pathogenesis of RA. Both HLA-related and non-HLA-related genetic areas of increased risk for RA were found [65]. The HLA-DR, HLA-DQ, and HLA-DP genes are part of the human leukocyte antigen (HLA) gene complex, which encodes the alpha and beta chains of the major histocompatibility complex (MHC) class II molecule. The MHC-II molecule is found on antigen-presenting cells and is responsible for presenting extracellular pathogens to T cells, resulting in an immune response. It is thought that the development of the disease is facilitated by the fact that HLA alleles of MHC-II bind and mispresent self-antigenic peptides to CD4+ T cells [66]. Based on available data, genetic associations of HLA genes with different subgroups of RA and in different populations are the most important contributors to genetic risk for RA [62]. These associations are primarily with HLA *DRB1* alleles that contribute at least 30% of the total genetic component of the disease [67]. The disease-associated alleles were revealed to have a conserved sequence of five amino acids, termed the “shared epitope” [66]. The “shared epitope” hypothesis suggests that certain alleles with this conserved sequence are associated with, and directly contribute to, the pathogenesis of RA by permitting the incorrect presentation of autoantigens to T cells by antigen-presenting cells [66]. The alleles of the “shared epitope” have been strongly associated with RA susceptibility and increased disease severity, as well as with an increased risk of developing extra-articular manifestations [68].

Several associations with RA outside the HLA locus were detected previously, including genetic alterations in *PTPN22*, *CTLA4*, and *PADI4* [69,70,71]. Currently, integrated data from GWASs and expression quantitative trait loci (eQTL) studies have shown that SNPs in PTPN22, CTLA4, and PADI4 are closely associated with their dysregulation, leading to autoimmune activation of B and T lymphocytes [58,72,73,74,75]. MYC, FOXO1, IL-20RA, TNFAIP3, OLIG3 gene, and miR-155 have been suggested as appropriate candidates for CRISPR-Cas9 treatment [76]. As the correlation between disease susceptibility and SNPs of certain genes discovered by computational analysis methods does not always accurately reflect the existence of such an association and needs to be confirmed, the main criterion for selecting candidate genes for genetic therapy is direct experimental confirmation of the involvement of the corresponding gene in the development of the autoimmune response in RA [62]. Using electrophoretic mobility shift assay (EMSA), Western blotting, chromatin conformation capture, and deletion knockouts of the candidate causal variant rs6927172 in HEK293T cells, rs6927172, located 140 kb upstream of TNFAIP3, was found to influence the expression of genes, including IL-20RA, TNFAIP3, and OLIG3, potentially involved in the autoimmune response, confirming its functional significance as a candidate for gene therapy [77]. Data based on ATAC-seq, Hi-C, Capture Hi-C, and nuclear RNA-seq analysis in stimulated CD4+ T cells suggest that MYC and FOXO1 genes may also be causal factors in RA and may therefore be candidates for gene therapy [78]. In the next study, after generating a miR-155 genome knockout in the RAW264.7 macrophage cell line, it was found that SHP1 was upregulated and the pro-inflammatory cytokine production process was disrupted, suggesting that genome editing of miR-155 may be a potential therapeutic strategy for RA [79]. Many other genes are also potential candidates for future editing using the CRISPR technique for the treatment of RA, once their functional significance has been experimentally confirmed [16,62]. For example, experimental data using the Jurkat T-cell line (ATCC TIB-152) clearly showed that edited SNPs in protein tyrosine phosphatase non-receptor types 2 and 22 (PTPN2/22) were associated with reduced gene expression, resulting in increased cell proliferation and activation and an overactive immune system, suggesting that they may be a potential candidate for future RA gene therapy [80]. Given the rapid development and improvement of genome editing technologies and the increasing amount of data on the genetics of RA, it is expected that the editing of genes associated with RA for therapeutic purposes will be successful in the near future.

### 2.3. Mesenchymal Stem Cell (MSC) Therapy

One of the cell therapy approaches, mesenchymal stem cell (MSC) therapy, allows for the restoration of immune homeostasis due to its effective manipulation of cells of the immune system. MSCs are adult multipotent stem cells with fibroblast-like morphology that have ability to differentiate into tissues of mesodermal origin, such as osteoblasts, chondrocytes, and adipocytes [81]. In addition, MSCs are involved in modulating the activity of the innate and adaptive immune systems by alleviating the proinflammatory phenotype and by promoting the anti-inflammatory phenotype, particularly through decreasing populations of DCs, macrophages, NK cells, B and T cells, and inducing the generation of Tregs [81]. MSCs can exert immunomodulatory effects either directly through cell–cell contact or indirectly through the secretion of soluble factors such as indoleamine 2,3-dioxygenase (IDO), hepatocyte growth factor (HGF), interleukin (IL)-10, transforming growth factor β (TGF-β), nitric oxide (NO), and tumor necrosis factor (TNF)-stimulated gene/protein 6 (TSG-6) [82]. Prostaglandin E2 (PGE2), a product of the cyclooxygenase-2 (COX-2) pathway, has been implicated as one of the key mediators in the immunomodulatory effect of MSCs [83]. For example, interaction between MSCs and Th17 cells has been shown to activate COX-2 signaling and release of PGE2 by MSCs, resulting in inhibition of Th17 cell proliferation, and can also lead to release of TGF-β by MSCs, inducing conversion of Th17 cells to Treg cells with upregulation of FoxP3 mRNA [84]. On the other hand, MSCs can prevent T-cell activation through a paracrine inhibitory effect by acting on the mitogen-activated protein kinase (MAPK) cascade of the NF-kB pathway and interfering with the activation of Toll-like receptors (TLRs) on DCs [85]. Cell–cell contact between MSCs and activated B cells has been shown to inhibit their differentiation into plasma cells by downregulating B-lymphocyte-induced maturation protein-1 (Blimp-1) through an unknown pathway, and can also induce the conversion of B cells into regulatory B cells that secrete IL-10 and suppress T-cell proliferation [86,87]. MSCs can be activated by pro-inflammatory signals when they interact with macrophages, creating two negative feedback loops [88]. In the first, MSCs increase PGE2 secretion by increasing the expression of COX2 and other components of the arachidonic acid pathway, thereby promoting the transition of macrophages to the M2 phenotype [89]. The second feedback loop occurs when MSCs secrete TSG-6, an anti-inflammatory molecule that binds to CD44 on macrophages and disrupts its interaction with TLR2, thereby reducing the inflammatory response induced by TLR2-mediated NF-kB signaling [90]. Such functional properties allow MSCs to potentially become used to treat RA. 

There are several MSC-based therapies for the treatment of RA that were studied in clinical trials: NCT01851070, NCT01663116, NCT03333681, NCT01873625, NCT01547091, and NCT02221258 [81,91,92,93,94,95,96]. Studies have shown that MSCs can significantly improve immune balance, inhibit T-cell activity, and regulate cytokine expression in the pathophysiological process of RA, as patients after MSC transplantation showed decreased levels of IL-1β, IL-6, IL-8, and TNF-α and increased levels of TGF-β1 and IL-10. In general, completed clinical trials have shown that infusion of MSCs is a safe and effective approach for the treatment of RA patients, with no serious adverse effects [91,92,93,94,95,96,97]. However, because MSC therapy is a more expensive procedure, it may be recommended for patients who are resistant to prolonged drug therapy with DMARDs or biologics and therefore may be susceptible to the side effects of this therapy. Examples of such side effects include infectious adverse events, embolism and thrombosis, neoplasia and gastrointestinal perforation events, and hepatotoxicity and skin reactions with JAK inhibitors [10]. Long-term use of biologic DMARDs causes immunogenicity and may also lead to an increased risk of serious infections due to their immunosuppressive effects [11,13,14]. Anti-TNF therapy has also been shown to increase the risk of cancer [17,18]. 

To increase the potential of MSCs in clinical applications for RA treatment and decrease the cost of the procedure, several strategies have been proposed [98]. For example, the combined application of MSCs and IL-10-producing Tregs was found to be more effective in preventing the development of CIA and suppressing inflammatory responses in joints in a mouse model than the uncombined therapy [99]. In another promising strategy, MSC cell preconditioning with high concentrations of proinflammatory cytokines may be used to enhance their immunosuppressive properties [100,101,102,103]. Additional prospective approaches are hypoxia and autophagy preconditioning in MSCs [104,105]. The combination of pro-inflammatory cytokines and hypoxia has also been found to have a synergistic effect [106]. Thus, given the immunomodulatory and immunosuppressive properties of this method, MSC therapy represents a promising approach in the treatment of RA. In the future, by using the above strategies to improve the therapeutic properties of MSCs, as well as reducing the cost of the procedure, it will be possible to obtain an effective and safe drug for widespread use in clinical practice.

### 2.4. Adoptive Treg Cells Therapy

Regulatory T cells (Tregs) are a subset of specialized CD4+ helper T cells defined by the expression of the IL-2 receptor α-chain (CD25) and the transcription factor Foxp3 [107]. Since the function of Tregs is to maintain immune tolerance by suppressing aberrant immune stimulation, adoptive Treg cell therapy for RA may be an important way to suppress autoimmune activity to prevent serious tissue and organ damage [108]. In adoptive Tregs therapy, autologous or allogeneic Tregs are isolated, ex vivo activated, expanded, and then infused to the patient [109]. The efficacy of adoptive Treg therapy was previously demonstrated in CIA in a mouse model, the most widely studied autoimmune model of RA [110]. Adoptively transferred Tregs have been shown in CIA models to rapidly accumulate in synovial tissue after injection and block CII-specific T-cell proliferation, significantly reducing disease severity and slowing disease progression [110]. Adoptive Treg therapy was then performed in a patient with SLE, and Treg accumulation and activation in the skin was associated with a marked attenuation of the IFNγ pathway and a reciprocal increase in the IL-17 pathway [111]. Adoptive transfer of Tregs also led to a similar decrease in Th1 and increase in Th17 in the tissue, accompanied by a reduction in the inflammatory process in a mouse model of Candida-induced skin inflammation [111]. There are also challenges to be addressed, such as the likelihood of Tregs converting into pathogenic cells [112]. It should be noted that the lack of specific antigen on the surface of Tregs makes them difficult to purify and increases the risk of Teff contamination [111]. Therefore, the presence of Treg cells isolated with high purity is a necessary condition for achieving good results when using the method of adoptive transfer of Treg cells to patients. These studies suggest that adoptive Treg cell therapy may be a viable approach for RA patients in the future, but further studies in different animal models and RA patients are needed to confirm the efficacy and safety of this therapeutic modality in clinical practice.

### 2.5. CAR-T Cell Therapy

Chimeric antigen receptor (CAR) T-cell therapy is a relatively new and rapidly evolving immunotherapy based on CAR-T cells, which are the patient’s own T cells genetically engineered to express a receptor on the T-cell membrane that can recognize specific target cell antigens [113]. Although originally developed for the treatment of cancer, some of the enthusiasm among researchers for using this approach in RA therapy is based on the successful use of CAR-T cells in the treatment of other autoimmune diseases, such as systemic lupus erythematosus (SLE) and systemic sclerosis (SSc) [114,115]. Anti-CD19 CAR-T cells have been used to treat patients with SLE and severe SSc, and in both cases significant improvement was achieved with no apparent side effects, demonstrating the efficacy, feasibility, and high potential of CAR-T cell therapy for use in clinical practice, including the potential treatment of other autoimmune diseases [114,115].

**Figure 2:**
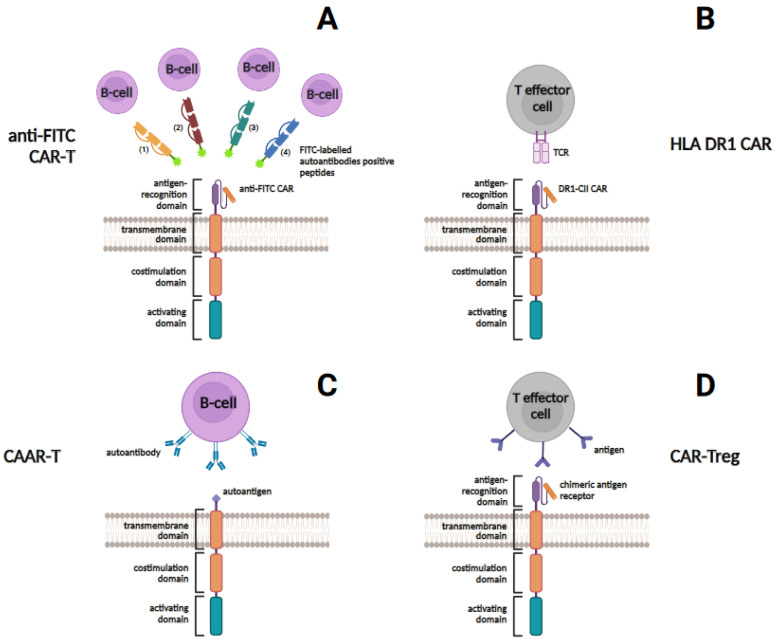
Four different approaches in CAR-T cell therapy for treatment of RA. (**A**) shows universal anti-FITC CAR-T cells. Using FITC-labeled autoantibody-positive citrullinated peptides, including citrullinated vimentin (1), citrullinated type II collagen (2), citrullinated fibrinogen (3), and tenascin C (4), as mediators, anti-FITC CAR-T cells can eliminate autoreactive B cells through peptide-mediated CAR-T cytotoxicity. (**B**) shows anti-DR1-CII CAR-T cells. In these CAR-T cells, the HLA-DRB1*01:01 (DR1) CAR molecule contains a covalently linked type II collagen autoantigenic peptide (CII) to specifically recognize and deplete autoimmune CD4+ T cells. (**C**) shows CAAR-T cell that recognizes autoantibody on the surface of the target B cell and induces a cytotoxic effect. (**D**) shows CAR-Treg that recognizes an antigen on the target cell and induces a regulatory response.

However, it is worth noting that, compared to SLE and SSc, the use of similar CAR-T cells targeting only one cell type is challenging in RA due to the heterogeneous population of autoreactive lymphocytes present in RA patients [116]. To work around this limitation, universal anti-fluoresceinisothiocyanate (anti-FITC) CAR-T cells have been proposed in tandem with FITC-labeled RA immunodominant peptides (Figure 2) [116]. This system allows CAR-T cells to target different types of autoreactive B-cell subsets according to a patient’s specific autoantigen profile [116]. A method originally proposed for the treatment of cancer has been used [117]. The approach was developed on the basis that one of the most specific serologic markers of RA is anticitrullinated protein antibodies (ACPA) [118]. To direct CAR-Ts to autoreactive B cells, four citrullinated peptides, such as citrullinated vimentin, citrullinated type II collagen, citrullinated fibrinogen, and tenascin C, were used as mediators conjugated with fluorescein isothiocyanate (FITC) [116]. Anti-FITC CAR-Ts were also prepared [119]. CAR-T cells have been shown to specifically recognize the FITC-labeled autoantigenic peptide epitopes and effectively target and eliminate autoreactive B cells expressing autoantibodies to citrullinated vimentin, citrullinated type II collagen, citrullinated fibrinogen, and tenascin C [116]. The main limitation of the study is that the therapeutic effect of CAR-T cells was only shown in in vitro experiments, and the stability of peptide-based mediators also raises questions [116]. However, the lack of cytotoxic activity in control experiments indicates the high selectivity of CAR-T cells and suggests the promise of this approach with further research and refinement.

Another proposed CAR-T cell design that could be used to treat RA is based on genetic data. As noted above, genetic susceptibility to RA is largely associated with certain HLA-DR alleles, including DR1 (DRB1*01:01) and DR4 (DRB1*04:01, *04:04, and *04:05), which share a common stretch of amino acids at positions 70 to 74 in the DRB1 chains, called the “shared epitope” [68,120]. The mechanism of association of these alleles with RA is that they bind and present an autoantigenic peptide to CD4+ T cells, contributing to the development of the disease [66]. Targeting pathogenic CD4+ T cells that recognize autoantigens presented by HLA-DR alleles is expected to be a highly effective treatment for RA [121]. To address this issue, a novel approach was developed in which the HLA-DRB1*01:01 (DR1) CAR molecule was engineered to include a CD3ζ activation domain, a CD28 signaling domain, and a covalently linked CII peptide (DR1-CII) to ensure specificity of action on autoimmune T cells (Figure 2) [121]. Stimulation of DR1-CII CAR-T cells with anti-DR antibodies has been shown to induce cytokine production, indicating that DR1-CAR functions as a chimeric molecule [121]. In vitro cytolytic activity assays of the CAR- T cells using cloned CD4+ T cells as targets showed that DR1-CII CAR-T cells selectively recognized and killed CD4+ T cells specific for the CII autoantigen [121]. The use of this type of CAR-T cells in experiments in the B6.DR1 mouse model showed a reduction in the severity of RA disease as well as a CII-specific autoimmune CD4+ T cell response, demonstrating the high potential of this approach for the treatment of RA [121]. However, clinical trials in patients are needed to confirm the efficacy and safety of this CAR-T cell therapy.

Two other types of CAR-T cells may also be useful in the management of RA, including chimeric autoantibody receptor T cells (CAAR-T cells) and chimeric antigen receptor in regulatory T cells (CAR-Tregs) (Figure 2) [122]. Unlike the scFv domain, CAAR has a selective affinity exclusively for autoantibodies on the surface of B cells, and CAAR-T cells bind to them and kill them through a cytotoxic effect [123]. For the first time, in experiments in a mouse model of PV, CAAR-T cells were successfully used to recognize the pemphigus vulgaris (PV)-specific autoantigen, desmoglein, and to eliminate autoreactive B cells [123]. Using CAAR-T cell therapy, successful targeted cell lysis has also been demonstrated in treating patients with autoimmune encephalitis [124]. It was hypothesized that CAAR-T cells bearing citrullinated antigens may be able to specifically eliminate anticitrulline B cells in RA [125]. Thus, the development and use of this CAR-T cell type for the treatment of RA is a distinct possibility in the future.

Since Tregs are usually suppressed in SAIDs, another effectual approach for the restoration of immune tolerance implies “switching” of T cell phenotype from cytotoxic to regulatory. This can be achieved with the help of CAR technology (Figure 2). CAR-Tregs are CAR-T cells that have been transformed into Tregs by the transduction of FOXP3, that regulates pathways involved in the formation and operation of regulatory T cells, coupled with CAR [126]. In addition, CAR-Tregs have been shown to be a source of immunomodulatory cytokines such as TGF-β, IL-10, and IL-35 and to promote apoptosis of Teff cells via granzyme B/A, Fas ligand and perforin, thus preventing the activity of Teff cells [127]. Citrullinated vimentin (CV), which is a specific antigen found exclusively in the inflamed synovial tissue of RA patients, has been suggested to be a potential target for CAR-T cell and CAR-Treg cell therapy [125,128]. It is also necessary to improve the stability of CAR-Tregs to avoid a rebound effect that could aggravate autoimmune reactions [125]. The above studies have identified the main directions for further development in the field of CAR-T cell therapy for RA. More comprehensive and extensive studies are needed to determine the model of CAR-T cell therapy that is most appropriate for the treatment of RA in terms of efficacy and safety. Given the current state of knowledge and experience in this area, CAR-T cell therapy may be a truly promising treatment that can take RA therapy to a new level, but serious side effects, such as the possible development of cytokine release syndrome, must be overcome, and the high cost of the procedure also limits the possibility of its widespread use.

## 3. Conclusions and Perspective

Treatment of RA, like any other autoimmune disease, faces challenges due to the variability of disease symptoms, lack of efficacy, and side effects of existing drugs. In addition, the same drugs can have different effects on different individuals, with some patients responding to certain drugs while others are resistant to them [7,15]. The emergence of a universal and effective treatment strategy for RA is still under development. However, with current medical technology and an ever-increasing body of knowledge, it is possible to overcome these challenges, and new therapeutic strategies have the potential to revolutionize the treatment of RA in the near future, offering qualitatively improved outcomes for the majority of patients.

With advancing technology and accumulating data, cell therapies represented by MSCs, Tregs, and CAR-T therapies have demonstrated preliminary therapeutic potential for the treatment of RA. Research in these areas represents a significant advance in targeted treatments for RA and provides direction for further research and development. However, careful preclinical and clinical studies are needed before these approaches can be applied in clinical practice, as many unresolved questions remain. Most studies in the field of MSC therapy have focused on assessing safety and efficacy, and some have been clearly insufficient in number of patients, ranging from one to three, while the composition, route of administration, dose, and frequency of use of MSCs have yet to be determined [91,92,93,94,95,96]. In addition, the immunosuppressive properties of MSCs were found to depend on the environment, in particular the presence of high levels of pro-inflammatory cytokines [100,101,102,103]. Adoptive therapy of Tregs has the potential for conversion to pathogenic cells [112]. The lack of a specific antigen on the surface of Tregs also makes them difficult to purify, increasing the risk of Teff infection [111,129]. With regard to CAR-based therapy, the best way to treat RA with CAR-T cells is not yet known. The main obstacle in the development of CARs for RA is the heterogeneity of the autoimmune lymphocyte cell population and therefore the heterogeneity of the targets for CAR-T cells, i.e., anti-CD19 cells, which have shown efficacy in the treatment of SLE and SSc, are not applicable to RA [116]. Several variants of CAR-T cells have been proposed for the treatment of RA and have shown preliminary efficacy. These studies provide direction for further research. However, in order for CAR-T therapy for RA to be used in the clinic, it is necessary to find the optimal type of CAR cells, confirm their safety and efficacy, and develop methods for producing these cells. The progress being made in this area gives us hope that all these issues will be successfully addressed. The method of treating RA by editing genes associated with RA is also attracting the attention of researchers and, after further development, may be used in practice. MicroRNAs, which are involved in the regulation of the immune response, also have therapeutic potential and can be used to diagnose and monitor the severity of the disease (Table 1) [34,35,39,40,41,42,44,45]. As always, there is a wealth of material to study the therapeutic effect of co-administration of therapeutic agents, both those already in clinical use and those under development. Given the great progress in the accumulation of knowledge in the field of RA therapy, treatment methods will be continuously improved, new therapeutic drugs will be developed, and each of the described new approaches to the treatment of RA may find its niche among the known methods and prove its viability in the near future.

## Figures and Tables

**Table 1:** miRNAs as potential biomarkers and therapeutic targets in RA.

miRNA	Targets	Potential to Treat RA
miR-181a	MTMR3	For diagnosis of RA
miR-371b	CSDE1	For diagnosis of RA
miR-483	IGF-1RB1, HDAC1, XIAP, IGF-1	For diagnosis of RA
miR-642b	-	For diagnosis of RA
miR-25	GZMA	For diagnosis of RA
miR-378d	IL-15	For diagnosis of RA
miR-23	CXCL12, NF-kB signaling	Biomarker for predicting and monitoring the outcome of RA therapy
miR-223	FLS	Biomarker for predicting and monitoring the outcome of RA therapy
miR-19b	JAK	Biomarker for predicting joint inflammation and response to JAK inhibitors
hsa-miR-21-5p	PTEN, PDCD4, RECK, HNRPK, JAG1, Bcl-2, PPARα	Might serve as a candidate for target therapy
miR-155	SOCS1 SHIP1	Might serve as a candidate for target therapy
miR-340-5p	STAT3	Might serve as a candidate for target therapy

## Abbreviations

ACPA	anticitrullinated protein antibody
Blimp-1	B lymphocyte-induced maturation protein-1
CAAR-T cells	chimeric autoantibody receptor T cells
CAR	chimeric antigen receptor
CIA	collagen-induced arthritis
CII	type II collagen
COX-2	cyclooxygenase-2
CRISPR	Clustered Regularly Interspaced Short Palindromic Repeats
CV	Citrullinated vimentin
DMARDs	disease-modifying antirheumatic drugs
FITC	fluoresceinisothiocyanate
FLS	fibroblast-like synoviocytes
GWAS	genome-wide association study
HGF	hepatocyte growth factor
HLA	human leukocyte antigen
IDO	indolamine-2,3-dioxygenas
IL-10	interleukin-10
JAK	Janus kinase
MAPK	mitogen-activated protein kinase
MHC	major histocompatibility complex
miRNA	MicroRNA
MSC	mesenchymal stem cell
MTMR3	myotubularin-related protein 3
NHL	non-Hodgkin’s lymphoma
NMSC	non-melanoma skin cancer
NO	nitric oxide
PBMC	peripheral blood monocytes
PGE2	prostaglandin E2
PV	pemphigus vulgaris
RA	rheumatoid arthritis
RA-FLS	fibroblast-like synoviocytes of rheumatoid arthritis
RF	rheumatoid factor
SLE	systemic lupus erythematosus
SNP	single nucleotide polymorphism
SSc	systemic sclerosis
TGF-β	transforming growth factor-β
TLR	Toll-like receptor
TNF	tumor necrosis factor
Tregs	regulatory T cells
TSG-6	tumor necrosis factor (TNF) stimulated gene/protein 6
